# Supplementary material for: Mechanisms governing the pioneering and redistribution capabilities of the non-classical pioneer PU.1
Source: Nat Commun. 2020 Jan 21;11:402. doi: 10.1038/s41467-019-13960-2 (PMC6972792; doi:10.1038/s41467-019-13960-2)
Supplement: Supplementary file 7 — Source data [file 41467_2019_13960_MOESM7_ESM.zip › Source_Data/Figure6/Figure6B_MotifScanOutput/ETS-specific/homerResults/motif12.similar.html]

motif12

## Information for motif12

C
T
A
G
A
C
T
G
A
C
T
G
G
T
A
C
A
C
T
G
A
C
T
G
C
T
G
A
T
C
G
A
T
G
A
C
G
A
C
T
  
Reverse Opposite:  

C
T
G
A
A
C
T
G
A
G
C
T
G
A
C
T
G
T
A
C
A
G
T
C
C
A
T
G
A
G
T
C
A
G
T
C
A
G
T
C
  

|  |  |
| --- | --- |
| p-value: | 1e-95 |
| log p-value: | -2.200e+02 |
| Information Content per bp: | 1.664 |
| Number of Target Sequences with motif | 2663.0 |
| Percentage of Target Sequences with motif | 34.79% |
| Number of Background Sequences with motif | 9373.9 |
| Percentage of Background Sequences with motif | 23.22% |
| Average Position of motif in Targets | 99.6 +/- 55.6bp |
| Average Position of motif in Background | 98.9 +/- 62.5bp |
| Strand Bias (log2 ratio + to - strand density) | 0.1 |
| Multiplicity (# of sites on avg that occur together) | 1.23 |
| Motif File: | file (matrix) reverse opposite |

### Similar de novo motifs found

|  |  |  |  |  |  |  |  |
| --- | --- | --- | --- | --- | --- | --- | --- |
| Rank | Match Score | Redundant Motif | P-value | log P-value | % of Targets | % of Background | Motif file |
| 1 | 0.890 | G T A C T A G C A T C G A G T C G A T C G T A C G T A C C A T G A G T C A G T C A G T C G T A C A T G C A G T C | 1e-94 | -218.149099 | 30.37% | 19.44% | motif file (matrix) |
| 2 | 0.901 | C T A G A C T G A C T G G T A C A C T G C A T G C T A G T C A G G A T C G A T C C T G A T A G C | 1e-83 | -193.063481 | 15.21% | 7.79% | motif file (matrix) |
| 3 | 0.860 | T C A G T A C G A T G C G A T C G T A C A G T C C A T G A G T C A G T C A G T C G A T C A G T C G A T C | 1e-75 | -174.695053 | 24.08% | 15.15% | motif file (matrix) |
| 4 | 0.859 | A C T G A C T G A C T G C G T A A C T G A C T G C T A G | 1e-58 | -135.429871 | 40.91% | 31.27% | motif file (matrix) |
| 5 | 0.887 | T C A G G A T C G C A T G T C A A T G C C A T G A G T C A G T C T A G C | 1e-56 | -129.480979 | 18.50% | 11.61% | motif file (matrix) |
